# Exome sequencing in dementia with Lewy bodies

**DOI:** 10.1038/tp.2015.220

**Published:** 2016-02-02

**Authors:** M J Keogh, M Kurzawa-Akanbi, H Griffin, K Douroudis, K L Ayers, R I Hussein, G Hudson, A Pyle, H J Cordell, J Attems, I G McKeith, J T O'Brien, D J Burn, C M Morris, A J Thomas, P F Chinnery

**Affiliations:** 1 Institute of Genetic Medicine, Newcastle University, Newcastle upon Tyne, UK; 2 NIHR Biomedical Research Unit, Newcastle upon Tyne Hospitals NHS Foundation Trust, Campus for Ageing and Vitality, Newcastle upon Tyne, UK; 3 Institute for Neuroscience, The Medical School, Newcastle University, Newcastle upon Tyne, UK

## Abstract

Dementia with Lewy bodies (DLB) is the second most common form of degenerative dementia. Siblings of affected individuals are at greater risk of developing DLB, but little is known about the underlying genetic basis of the disease. We set out to determine whether mutations in known highly penetrant neurodegenerative disease genes are found in patients with DLB. Whole-exome sequencing was performed on 91 neuropathologically confirmed cases of DLB, supplemented by independent *APOE* genotyping. Genetic variants were classified using established criteria, and additional neuropathological examination was performed for putative mutation carriers. Likely pathogenic variants previously described as causing monogenic forms of neurodegenerative disease were found in 4.4% of patients with DLB. The *APOE* ɛ4 allele increased the risk of disease (*P*=0.0001), conferred a shorter disease duration (*P*=0.043) and earlier age of death (*P*=0.0015). In conclusion, although known pathogenic mutations in neurodegenerative disease genes are uncommon in DLB, known genetic risk factors are present in >60% of cases. *APOE* ɛ4 not only modifies disease risk, but also modulates the rate of disease progression. The reduced penetrance of reported pathogenic alleles explains the lack of a family history in most patients, and the presence of variants previously described as causing frontotemporal dementia suggests a mechanistic overlap between DLB and other neurodegenerative diseases.

## Introduction

Dementia with Lewy bodies (DLB) is the second most common form of dementia. It affects 5% of the population over 75 years of age,^1^ and has a greater impact on healthcare provision than Alzheimer's disease (AD).^2^ The neuropathological hallmark of DLB is widespread α-synuclein-positive neuronal inclusions (Lewy bodies and Lewy neurites) and in addition this is often associated with amyloid deposition.^3^ Siblings of affected individuals have a 2.3-fold increased risk of developing the disorder,^4^ but little is known about the genetic aetiology of the disease. Although genetic variants in *APOE,*^5^ *GBA,*^6^ *SNCA* and *SCARB2* (ref. 7) have been associated with an increased risk of DLB, only a few families have been described with more than two first-degree relatives,^8^ and no single highly penetrant gene defects have been shown to cause familial forms of the disorder. Using exome sequencing in 91 autopsy-confirmed cases, here we determined whether confirmed or putative pathogenic mutations in genes in known neurodegenerative disease genes are found in patients with DLB.

## Materials and methods

### Subjects and sample preparation

**Figure 1:**
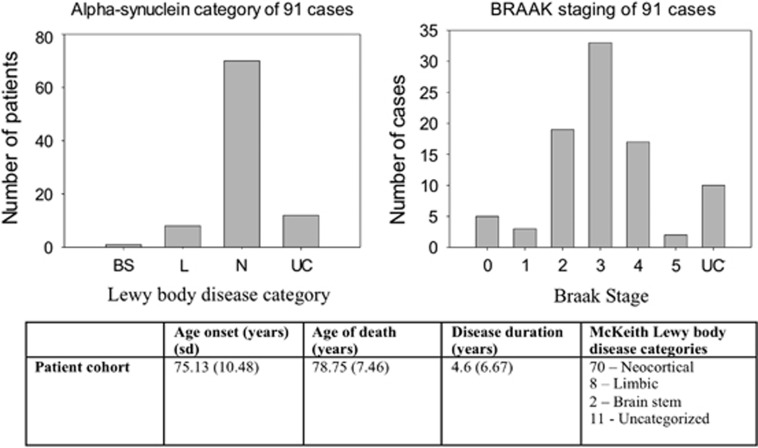
Clinical and pathological characteristics of the 91 dementia with Lewy body (DLB) cases. Top left: frequency of each pathological category (BS, brain stem; L, limbic; N, neocortical; UC, unclassified). Top right: BRAAK neurofibrillary tangle stage of patients (UC, unclassified). Bottom: table of the clinical and pathological data for all the 91 cases of DLB. Data are mean (s.d.). Motor features were defined by documented evidence of a Parkinsonian movement disorder by an assessing clinician.

We studied 91 post-mortem cases conforming to both the clinical and post-mortem diagnostic criteria for DLB.^3^ Two patients were first-degree relatives (mother and daughter) and two patients were siblings (brothers). The remaining 87 patients had no recorded family history of neurodegenerative disease. Age of onset, disease duration, age of death, neuropathological subtype of Lewy body disease according to McKeith/Newcastle criteria^3^ and Braak neurofibrillary tangle stage were recorded^9^ (Figure 1). In addition, we assessed Lewy body Braak stages,^10^ Aβ phases^11^ and stages of cerebral amyloid angiopathy.^12^ Of note, none of the cases showed intracytoplasmic TAR DNA-binding protein 43 (TDP-43) inclusions indicative for frontotemporal lobar degeneration associated with TDP-43 pathology, nor were there neuropathological features consistent with other types of frontotemporal lobar degeneration (see additional Supplementary Methods).

### DNA extraction and exome sequencing

DNA was extracted from cerebellum in all the cases. Illumina TruSeq 62 Mb exome capture and sequencing (Illumina Hiseq2000, 100 bp paired-end reads) was performed as described (see additional Supplementary Methods).

Known disease genes were defined as those previously shown to cause monogenic forms of Parkinson's disease (PD), AD, frontotemporal lobar dementia and amyotrophic lateral sclerosis (Table 1). Variants were selected with a minor allele frequency of <0.01 international reference databases. Variants were defined as (1) pathogenic, (2) likely pathogenic, (3) of uncertain significance or (4) benign according to American College of Medical Genetics criteria^13^ (Table 1).

For completeness, exonic variants in genes previously associated with DLB (*GBA, APOE, SNCA* and *SCARB2*),^5, 6, 7^ AD (*APOE, TREM2*)^14^ or PD (*LRRK2, GBA*)^15^ were also identified in DLB cases and compared with 93 in-house unrelated disease control exomes.

## Results

The mean exome sequencing base coverage depth was 84-fold (s.d.=13) in the 91 DLB cases and 76-fold (s.d.=12) in the 93 controls. There was no difference in the proportion of the exome target covered at >30-fold depth between DLB cases and controls (DLB 84%, s.d.=5; controls 84%, s.d.=3, *P*=0.588).

### Known mendelian disease genes

A total 18 rare heterozygous mutations in 25 patients were observed in genes previously shown to cause autosomal dominant forms of neurodegeneration (Tables 1, 2 and Supplementary Table S1). Three of these variants have been described in patients with AD, PD or frontotemporal lobar degeneration and amyotrophic lateral sclerosis (Patient A:*PSEN2* p.D439A;^16, 17^ B:*CHMP2B* p.I29V;^18^ and C:*SQSTM1* p.A33V,^19, 20^). In two additional cases (Patient E:*EIF4G1* p.M1134V and F:*SQSTM1* p.P27L), variants in known disease genes affecting highly conserved residues and predicted to be pathogenic by *in silico* software algorithms, were deemed of uncertain significance. Two patients also had variants of uncertain significance in *GIGYF2,* which is also implicated in PD (H:*GIGYF2* p.S66T; G:*GIGYF2* p.S1029C, Table 2). In genes causing autosomal recessive PD, AD or frontotemporal dementia and amyotrophic lateral sclerosis, only one rare compound heterozygous mutation in *PARK2* was seen (Patient D, p.R275W/p.G430D).

Only patient A had a relevant family history (father affected—deceased and no tissue/DNA available). A clinical description of these cases is shown in the Supplementary Information. All showed typical DLB pathology with cortical LB being present and moderate AD pathology (Table 2).

The mean age at the presentation for the four cases with previously described pathogenic mutations (Patients A–D) was 78.25 years (s.d.=8.05). Motor symptoms developed in three cases (Patient A, B and D) at a mean of 1.33 years (s.d.=0.58) after the onset of cognitive symptoms. When patients E and F were included, the mean age of onset was 78.6 (s.d.=6.68), with motor symptoms developing in four patients (A, B, D and E), and a mean disease duration of 2.3 (s.d.=1.16) years.

### Major risk alleles

*GBA*, *TREM2* and *LRRK2* had >80% coverage at 30-fold depth in both DLB cases and controls. *APOE* coverage was poor (DLB, 46.2% controls 48.7% at 30-fold depth) and was therefore genotyped independently (see additional Supplementary Methods). After removing the previously described pathogenic alleles, *APOE* ɛ4 was significantly associated with DLB compared with controls (*n*=87, *P*=0.0001, Table 3). Ten DLB cases had one of five heterozygous *GBA* variants, compared with only three controls (*P*=0.043). Two *GBA* variants known to be risk factors for PD (p.L370P and p.N296S) were seen only in four patients and no controls. Two patients had variants in *SCARB2* compared with six controls, and no *SNCA* variants were seen. There was no association between DLB and variants in *SCARB2*, *LRRK2* or *TREM2* (Supplementary Table S2).

**Figure 2:**
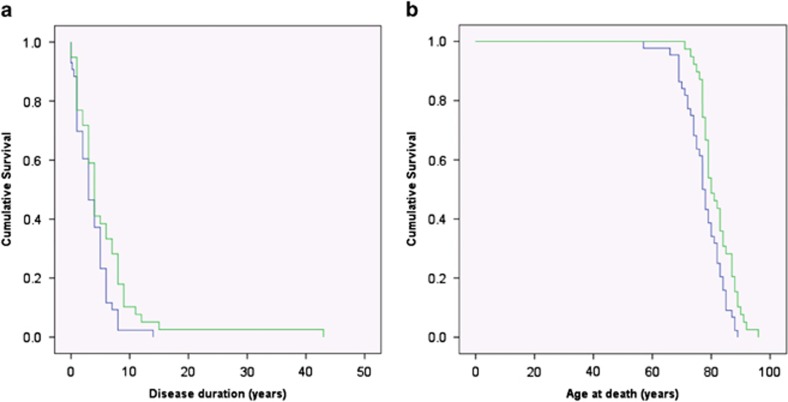
Kaplan–Meier survival curves for DLB patients by APOE allele. Kaplan–Meier survival curves for DLB patients by APOE allele carrying at least one *APOE* ɛ4 allele (*n*=43, blue line), compared with non-*APOE* ɛ4 carriers (*n*=39, green line). Despite there being no significant difference in the age of onset of the DLB (see Results), *APOE* ɛ4 carriers (**a**) lived for a shorter period of time following diagnosis (*P*=0.036, log rank, Mantel–Cox test), and thus (**b**) died at a younger age (*P*=0.005, log rank, Mantel–Cox test) that non-*APOE* ɛ4 carriers. DLB, dementia with Lewy body.

Although there was no difference in the age of onset of DLB in *APOE* ɛ4 allele carriers when compared with non-*APOE* ɛ4 allele carriers (*P*=0.227), the *APOE* ɛ4 allele carriers had a shorter disease duration following diagnosis (*P*=0.036), and died at an earlier age (*P*=0.005) than non-APOE ɛ4 carriers (Figure 2, Table 3). There was no association between the presence of variants in *GBA, SNCA, SCARB2, LRRK2*, *PARK2* or *ATP13A2* and age of onset, disease duration, age of death, neurofibrillary Braak stage or the presence of motor symptoms.

## Discussion

Exome sequencing of 91 cases of pathologically confirmed DLB identified four patients harbouring previously described pathogenic mutations neurodegenerative disease genes based on current diagnostic criteria (*PSEN2, CHMP2B, SQSTM1, PARK2*); possible pathogenic mutations in two (*EIF4G1* and *SQSTM1*); and two further cases with mutations in *GIGYF2,* which has previously been associated with autosomal dominant PD. The central question is: are these variants causing DLB, or are they co-incidental findings? The role of *GIGYF2* in PD remains contentious,^21^ and the p.D439A variant in *PSEN2* may have incomplete penetrance,^17^ and is thus found in control databases along with the *CHMP2B* and *SQSTM1* variants. Providing definitive proof of pathogenicity is therefore challenging, and there are arguments in both directions.

On one hand, the variants detected in *PSEN2, CHMP2B, SQSTM1* and *PARK2* are exceptionally rare in the general population.^22^ Given the clinical, pathological and mechanistic overlap between DLB and the neurodegenerative disorders where these disease genes were first described, it is plausible that they are contributing to the neuropathology. For example, in families with familial AD due to *PSEN2* mutations, up to 64% of cases have extensive Lewy body deposition at autopsy.^23^ The CHMP2B protein has been shown to be found in association with Lewy bodies in post-mortem cases of DLB,^24^ and SQSTM1 deficiency has been shown to enhance α-synuclein accumulation in mice.^25^ The *SQSTM1* p.A33V variant was previously described in five cases of frontotemporal dementia.^19, 20^ Recently, this allele was also detected in a patient with young-onset AD.^26^ Although seen in 0.0012% of controls, the p.A33V variant has now been seen in 8/1060 (0.007%) of patients with a neurodegenerative disease (including our study)^19, 20,26^ suggesting a broad association with neurodegenerative disorders (*P*=0.0037, chi squared with Yate's correction). These findings support the notion that rare, incompletely penetrant pathogenic alleles cause overlapping syndromes of neurodegeneration, perhaps explaining why previously ascribed variants for frontotemporal dementia were also found in our DLB cases. Pathogenic mutations with a reduced penetrance will also be detected in healthy individuals (as for *PSEN2* p.D439A^17^), and their presence in a control cohort does not preclude their potential to cause disease.^22^ This may explain why none of the four patients harbouring established pathogenic mutations reported a relevant family history.

On the other hand, the clinical and pathological phenotype of these five cases was wholly typical of DLB: how can this be reconciled with known pathogenic compound heterozygous mutations in *PARK2,* which typically presents with dystonia in early adult life? These findings highlight the challenges of using exome or whole-genome sequencing in a clinical context: is rare pathogenic mutation in a known disease gene more likely to be causing a variant phenotype, or is the phenotype so unusual that the variants must be a co-incidental finding? This will be difficult to resolve in individual cases, but the ongoing reporting of rare putative disease alleles, linked to rich phenotypic data, is an essential step in generating global data sets, which will ultimately provide definitive evidence of pathogenicity.^22^

Although the size of our study cohort limited the potential to discover new disease genes and risk loci, and did not include exclusion of repeat expansions such as *C9orf72*, we saw enrichment of *GBA* alleles and *APOE* ɛ4 alleles in DLB. In total, 48 patients (55.2%) possessed an *APOE* ɛ4 allele, with 5 (5.7%) having a variant in GBA, together with four (4.4%) having likely pathogenic alleles (potentially with incomplete penetrance). Therefore, 62.6% of patients harbour a risk factor or potentially pathogenic allele. This could explain why DLB is a relatively common disorder in the population, with an increased risk of disease within families, but few pedigrees suggestive of highly penetrant alleles. Finally, the association between *APOE* genotype and clinical progression has, to our knowledge, not been previously described, and has implications for cohort stratification in treatment studies.

## Figures and Tables

**Table 1:** Genes causing monogenic forms of PD, AD, FTLD-ALS, which were analysed for rare protein altering mutations in patients

*Inheritance*	*Disease*		
	*PD*	*AD*	*FTLD-ALS*
Autosomal dominant	*SNCA* *LRRK2* *UCHL1* *GIGYF2* *Omi/HTRA2* *EIF4G1*	*APP* *PSEN-1* *PSEN2*	*C9orf72* *SOD1* *MAPT* *PGRN* *TARDBP* *OPTN* *ANG* *CHMP2B* *SQSTM1* *FUS* *VCP*
Autosomal recessive	*PARK2* *PINK1* *ATP13A2* *PLA2G6* *FBX07* *DJ-1*		*OPTN*

Abbreviation: AD, Alzheimer's disease; ALS, amyotrophic lateral sclerosis; FTLD, frontotemporal lobar degeneration; PD, Parkinson's disease.

**Table 2:** The frequency of potentially pathogenic variants in DLB cases and controls

*Patient*	*Pathogenicity*	*Allele information and protein alteration*									*Functional prediction of variant*				*Neuropathology*					*Pathogenicity*
		*Gene*	*Chromosome*	*Position*	*R/V*	*Predicted protein change*	*Previously reported phenotype*	*MAF ESP 6500*	*MAF 1000G*	*ExAC MAF*	*SIFT*	*PolyPhen2*	*Mutation-taster*	*CADD score (scaled)*	*NFT Braak stage*	*Braak PD stage*	*Aβ phase*	*TDP-43*	*CAA*	*ACMG criteria*
A	P	*CHMP2B*	3	87289899	A/G	p.I29V	FTLD	0.00015	—	0.0001237	T	N	D	14.1	4	5/6	4	+ve CA1	2	(1) Same amino acid as previously reported (PS1) (2) Well established functional studies show a deleterious effect (PS3)
B	P	*PARK2*	6	162206852	G/A	p.R275W	PD	0.001999	0.0005	0.00206	D	D	D	33	4	6	3	NT	—	(1) Same amino acid as previously reported (PS1) (2) Well established functional studies show a deleterious effect (PS3)
B	P	*PARK2*		161771240	C/T	p.G430D		0.000231	—	0.0001076	D	D	D	34						
C	LP	*PSEN2*	1	227083249	A/C	p.D439A	AD	0.00015	—	0.00003764	D	D	D	26.9	4	6	4	NT	2	(1) Same amino acid as previously reported variant (PS1) (2) Multiple lines of computational evidence (PP3) (3) Missense with low rate of benign variability (PP2)
D	LP	*SQSTM1*	5	179250906	C/T	p.A33V	FTLD	0.000769	0.0018	0.001523	T	N	N	11.9	3/4	6	3	+ve CA1	2	(1)Same amino acid as previously reported (PS1)
E	US	*EIF4G1*	3	184046450	A/G	p.M1134V	U	0.00015	—	0.0002224	D	D	D	26.3	3	5/6	2	+ve CA1	2	(1) Computational evidence supports a deleterious effect
F	US	*SQSTM1*	5	179250888	C/T	p.P27L	U	0.00008	—	0.00003349	T	D	N	12.8						(1) Computational evidence supports a deleterious effect
G	US	*GIGYF2*	2	233709083	C/G	p.S1029C	U	0.00123	—	0.0007833	D	D	D	23.2	5	6	4	−ve	2	(1) Computational evidence supports a deleterious effect
H	US	*GIGYF2*	2	233655546	G/C	p.S66T	U	0.00008	—	0.0001813	T	D	D	23.7	4	5	3	NT	2	(1) Computational evidence supports a deleterious effect

Abbreviations: ACMG, American College of Medical Genetics; AD, Alzheimer's disease; CA1, CA1 division of the hippocampus; CAA, cerebral amyloid angiopathy; DLB, dementia with Lewy body; FTLD, frontotemporal lobar degeneration; MAF, minor allele frequency; PD, Parkinson's disease; R, reference allele; U, unknown or not described; V, variant allele.

The number of patients covered at >30-fold sequence depth, and the number of case and control patients carrying each mutation is shown. Functional predictions were performed by SIFT, PolyPhen2 and MutationTaster. Variants were classified as: (1) pathogenic, if the same alleles had previously been described in patients with neurodegenerative disease; (2) likely pathogenic, if the alleles were in previously known neurodegenerative disease genes and *in silico* predictions supported a pathogenic role; and (3) possibly pathogenic, if *in silico* predictions supported a pathogenic role, and the gene had previously been associated with a Mendelian neurodegenerative disease. See Supplementary Material for citations. Neuropathology scores according to existing accepted diagnostic criteria as outlined in Supplementary Methods are shown.

**Table 3:** APOE genotype of all cases (excluded confirmed pathogenic variants) and controls

*Study size*		*APOE genotype*						
		*4/4*	*3/3*	*2/2*	*4/3*	*3/2*	*4/2*	*ɛ4 carrier*
Controls	93	1	54	2	24	12	0	25
DLB patients	87	3	33	0	45	6	0	49
								
*P*-value		0.35	0.0076	0.50	0.0004	0.22	1.0	0.0001

Abbreviation: DLB, dementia with Lewy body.

Comparison between groups (patients *n*=87, controls *n*=91) performed by Fisher's exact test. *APOE* ɛ4 carrier determined by the presence of at least one *APOE* ɛ4 allele.
