# Supplementary material for: Exome sequencing in dementia with Lewy bodies
Source: Transl Psychiatry. 2016 Feb 2;6(2):e728–. doi: 10.1038/tp.2015.220 (PMC4872424; doi:10.1038/tp.2015.220)
Supplement: Supplementary Information [file tp2015220x1.docx]

Supplementary Information

MJ Keogh *et al.* Exome sequencing in Dementia with Lewy bodies

Supplementary methods Page 2

Clinical and pathological descriptions, discussion of mutation pathogenicity Page 6

Discussion of mutations unlikely to be a cause of monogenic DLB Page 10

Supplementary tables Page 12

Supplementary references Page 15

**Supplementary Methods**

**DNA extraction and sequencing**

All tissue extraction was approved by the relevant local ethics committee. Genomic DNA was extracted from 200mg of cerebellum, fragmented and enriched using Illumina TruSeq™ 62Mb exome capture and sequenced (Illumina Hiseq2000, 100bp paired-end reads). Bioinformatic analysis was performed using an in-house algorithm including alignment (human reference genome hg19, UCSC) using Burrows-Wheeler Aligner (BWA) [^1^](#_ENREF_1), reformatting using SAMtools [^2^](#_ENREF_2), and variant calling using VarScan [^3^](#_ENREF_3) and Dindel [^4^](#_ENREF_4). Subsequent analysis was restricted to on-target homozygous, heterozygous and compound heterozygous variants defined by stringent coverage criteria (>30-fold coverage). Predicted functional effects were determined using Mutation-Taster [^5^](#_ENREF_5), SIFT [^6^](#_ENREF_6) , Polyphen2 [^7^](#_ENREF_7) and the PHRED-scaled CADD score [^8^](#_ENREF_8)

**Systematic review to identify known monogenic disease genes.**

To determine genes which can cause monogenic forms of Alzheimer’s disease (AD), Parkinson’s disease (PD), Frontotemporal dementia (FTD), Amyotrophic lateral sclerosis ALS), and Dementia with Lewy bodies (DLB), we searched PubMed with the terms ‘monogenic AND Alzheimer’s’, ‘monogenic AND Parkinson’s’, ‘monogenic AND frontotemporal dementia, ‘monogenic AND FTD’, ‘monogenic AND amyotrophic lateral sclerosis’, ‘monogenic AND ALS’, ‘monogenic AND motor neuron disease’ ‘monogenic and Dementia with Lewy bodies’, ‘monogenic AND DLB’, ‘inherited AND Alzheimer’, ‘inherited AND Parkinson’s’, ‘inherited AND frontotemporal dementia’, ‘inherited AND FTD’, ‘inherited AND Dementia’, ‘inherited AND ALS’.

Firstly genes conclusively shown to cause monogenic forms of disease were selected. Heterozygous variants with evidence of more than 2 first degree affected family members carrying the mutation, which segregated with disease, and not seen in controls were first selected (*SNCA* [^9^](#_ENREF_9)^,^ [^10^](#_ENREF_10), *LRRK2* [*^11^*](#_ENREF_11) *C9orf72* [*^12^*](#_ENREF_12)*, SOD1* [*^13^*](#_ENREF_13)*, PSEN1* [*^14^*](#_ENREF_14)*, PSEN2* [*^15^*](#_ENREF_15)*, APP* [*^16^*](#_ENREF_16)*, PGRN* [*^17^*](#_ENREF_17)*, MAPT* [*^18^*](#_ENREF_18)*, CHMP2B* [*^19^*](#_ENREF_19)*, FUS* [*^20^*](#_ENREF_20)*, ANG* [*^21^*](#_ENREF_21)*, VCP* [*^22^*](#_ENREF_22)). Secondly those with 2 first degree relatives were also included (*SQSTM1* [*^23^*](#_ENREF_23)*,UCHL1* [^24^](#_ENREF_24)), and finally genes in which non-synonymous mutations have been shown in some studies to be more common in cohorts of sporadic patients than controls (*GIGYF2* [^25^](#_ENREF_25), Omi/*HTRA2* [^26^](#_ENREF_26)).

Homozygous or compound heterozygous mutations in genes identified through autozygosity mapping or linkage studies and which have been replicated in large cohorts of sporadic patients were included (*parkin* (PARK2) [^27^](#_ENREF_27), *PINK1* (PARK6) [^28^](#_ENREF_28) and *DJ-1* [^29^](#_ENREF_29)) and those identified in isolated families but not yet replicated in large cohorts (*ATP13A2* [^30^](#_ENREF_30) , *PLA2G6* [^31^](#_ENREF_31), *FBX07* [^32^](#_ENREF_32)).

To determine moderate risk factors, articles about genetic risk factors were identified through specific searches in AlzGene for genome-wide association studies and other large scale association studies for Alzheimer’s disease, PubMed for established risk factors for DLB, and PubMed and PDgene for genes associated with Parkinson’s disease. Genes with an established Odds ratio (OR) of disease of 3.0 or over were classified as moderate risk factors, and those with a lower odds ratio as risk loci. The final list of papers was generated between Jan 1988 and September 2013.

***APOE* genotyping by restriction fragment length polymorphism analysis**

A 318bp fragment of the *APOE g*ene which spanned both *APOE* polymorphic sites was amplified in a 50μl reaction using 25ng of DNA extracted from brain tissue for each of the 87 patients and 93 age matched controls. Two primers were used in the amplification process: Upstream primer E2 mut (5’ACT GAC CCC GGT GGC GGA GGA GAC GCG TGC) and the downstream primer (5’ TGT TCC ACC AGG GGC CCC AGG CGC TCG CGG). The E2 mut primer differs from the genomic sequence at one position to create an additional AflIII recognition site in the amplified fragment. Reactions were incubated at 94 °C for 3 minutes, followed by 40 cycles of amplification (94°C for 10 sec, 65°C for 30sec, 72°C for 30 sec) and incubated at 72°C for 7 minutes.

Restriction digests were subsequently performed; 10μl of the amplified product were mixed with 5 units of AflIII (New England Biolabs) and the buffer supplied by the manufacturer (NEB 3), and a further 10μl of amplified product were mixed with 10 units of HaeII (New England Biolabs) in the buffer supplied by the manufacturer (NEB4) overnight. Bands were subsequently visualised with a 2% agarose gel, and genotype determined as previously described [^33^](#_ENREF_33).

**Neuropathology**

For immunohistochemistry, 6µm paraffin sections were de-waxed and rehydrated through graded alcohols to water then endogenous peroxidase activity was blocked with 0.3% hydrogen peroxide in absolute methanol for 20 min. After washing in tap water, antigen retrieval was performed. Primary antibodies used were mouse monoclonal AT8 against a phosphorylated epitope in microtubule associated protein tau (Insight Biotechnology, Mamhead, UK; 1:500 dilution), mouse monoclonal anti-Aβ (4G8; Dako, High Wycombe, UK; 1:1500 dilution), anti-α-synuclein (Novocastra Laboratories, Newcastle, UK, 1:100 dilution), and rabbit anti-mouse TDP43 (TARDBP, Protein Tech 1:1000 dilution). For AT8, sections were immersed in 0.1 M citrate buffer pH 6 and placed in a microwave and the microwave run on full power until the citrate buffer had just begun to boil. At this point, the power was reduced and the sections were simmered for 10 minutes. For anti-Aβ, TDP43, and α-synuclein immunohistochemistry, formic acid pre-treatment was performed. Sections were immersed in 98% formic acid for 3 minutes and then rinsed in tap water. The sections were then rinsed three times, for 5 min each, in phosphate-buffered saline (PBS) and in the case of α-synuclein, placed in a decloaker containing 0.1M EDTA, pH8.0 and heated for 3 minutes. Following cooling of the sections, non-specific protein binding was blocked by incubation in 1% serum for 40 minutes, sections were then incubated with the primary antibody at room temperature for 1 hour. After rinsing (3 x 5 min in PBS), the antibody was detected using using a Menapath HRP polymer detection kit (Menarini diagnostics, Berkshire, UK). The sections were then washed and lightly counterstained with haematoxylin, dehydrated through graded alcohols, cleared in xylene and mounted in DPX mountant.

Neuropathological assessment was performed according to published criteria and included staging of neurofibrillary tangles/ neuropil threads (NFT Braak stages [^34^](#_ENREF_34)), Lewy bodies/ Lewy neurites (LB Braak stages and McKeith/Newcastle criteria [^35^](#_ENREF_35)), parenchymal Aβ pathology (Aβ Phase [^36^](#_ENREF_36)) and assessment of cerebral amyloid angiopathy (CAA [^37^](#_ENREF_37)) . Of note, in no cases did we observe TDP-43 positive inclusions in the cytoplasm or nucleus. In addition, no TDP-43 positive threads in the neuropil were seen. Hence, none of our cases showed features of FTLD-TDP-43 (Table 2, main text).

**Clinical and pathological descriptions**

Patient A: (*PSEN2* p.D439A). This patient developed problems with short-term memory aged 70. Over the following 6 months short-term memory loss progressed, and they developed visual hallucinations with significant fluctuations, and marked paranoia. A year after symptoms started they developed rapidly progressive Parkinsonism. The patient died 18 months after symptoms began aged 71.

Neuropathological examination, as outlined in Table 2 (main text) showed a NFT Braak stage 4, Aβ Phase of 4, neocortical Lewy body disease/Lewy body Braak stage 6, CAA stage of 2, and no TDP-43 positive intracytoplasmic inclusions.

A review of clinical notes showed that the patient’s father also had a rapidly progressive dementia in his early 70s and had been diagnosed with Parkinson’s disease shortly afterwards, but no further information was available in medical records.

The *PSEN2* p.D439A mutation has previously been reported in a Spanish Family with early onset Alzheimer’s disease (EOAD) but in the absence of any demonstrable movement disorder and without visual hallucinations [^38^](#_ENREF_38). The variant also was present in association with a heterogeneous clinical phenotype within 2 cases from an extended Italian Pedigree [^39^](#_ENREF_39).

Patient B: (*CHMP2B* p.I29V). This patient presented aged 87 to psychiatry with an acute onset of delusions, paranoia and depression and was originally diagnosed with a psychotic depression. One year later he developed a marked tremor and Parkinsonism which progressed over the next 6 months. He died 6 months later from throat cancer, 18 months after symptoms began.

Additional neuropathological examination, as outlined in Table 2 (main text) showed an NFT Braak stage of 4, Aβ Phase of 4, LB Braak stage of 6, CAA stage of 2 and TDP-43 positive intracytoplasmic inclusions limited to sector CA1 of the hippocampus. The p.I29V mutation has previously been described in a patient in their mid-60s who developed a frontal lobe syndrome before the slower development of ALS [^40^](#_ENREF_40).

Patient C: (*SQSTM1* p.A33V). This patient presented aged 73 with word finding difficulties and attentional deficits. Six months later he developed complex visual hallucinations, and over the following 2 years fluctuating episodes of anxiety and aggression. An EEG was diffusely slow, and a CT scan of his head showed marked generalised atrophy. There were no motor features. He died of a stroke aged 77, four years after onset. The p.A33V mutation has previously been identified in 3 patients with FTD-ALS, and was not seen in 724 controls [^41^](#_ENREF_41)^,^ [^42^](#_ENREF_42). Patients in this cohort also presented with variable clinical phenotypes ranging from young onset limb dominant ALS, to late onset slowly progressive frontotemporal dementia.

Neuropathological examination, as outlined in Table 2 (main text) showed a NFT Braak stage 3 Aβ Phase 3, Neocortical Lewy body disease/LB Braak stage 6, CAA stage of 2, and TDP-43 positive intracytoplasmic inclusions limited to sector CA1 of the hippocampus.

Patient D (*PARK2* p.R275W and p.G430W). The patient carrying this compound heterozygous mutation presented aged 82 with visuospatial difficulties, in particular interpreting maps together with difficulty with short-term memory, and significant social apprehension and paranoia. Over the next 2 years they developed very fluctuant but severe paranoia and delusions, together with a marked deterioration in self-care. Two years after symptoms began he developed a marked tremor and Parkinsonian gait resulting in him becoming non-ambulatory over a period of a year. He died aged 84, 1 year after motor symptoms developed.

Neuropathological examination as outlined in Table 2 (main text) showed a NFT Braak stage 4, neocortical Lewy body disease/LB Braak stage 6, Aβ Phase of 3, no CAA and no TDP-43 positive intracytoplasmic inclusions.

Compound heterozygous mutations involving the p.R275W mutation have been previously described [^43^](#_ENREF_43)^,^ [^44^](#_ENREF_44), and the p.G430D mutation causes disease in both homozygous and compound heterozygous state [^44^](#_ENREF_44).

Patient E (*EIF4G1* p.M1134V). The patient developed symptoms at the age of 76, initially describing difficulties with short-term memory followed by visuospatial problems such as inability to complete puzzles, with a clear fluctuant pattern. Approximately 18 months after the onset of cognitive symptoms, and aged 78, he developed a unilateral tremor, and some mild bradykinesia. Over the next 2 years his motor symptoms remained very mild, but there was a dramatic deterioration in cognitive function. He died a year later aged 79.

Neuropathological examination, as outlined in Table 2 (main text) showed an NFT Braak stage of 3, Aβ Phase of 2, neocortical Lewy body disease/LB Braak stage of 6, CAA stage of 2 and TDP-43 positive intracytoplasmic inclusions limited to sector CA1 of the hippocampus.

Patient F (*SQSTM1* p.P27L). This patient presented aged 84 with a 1-year history of progressive disorientation, aggression and marked paranoia. Prior to this she had been living independently. Over the next 6 months she developed visual hallucinations and worsening aggression requiring neuroleptic medication and admission to a nursing home. There were no motor features. She died 14 months after the onset of disease from bronchopneumonia aged 85.

Neuropathological examination, as outlined in Table 2 (main text) showed an NFT Braak stage of 4, Aβ Phase of 1, neocortical Lewy body disease/LB Braak stage of 6, no CAA and no TDP-43 positive intracytoplasmic inclusions.

Patient G (*GIGYF2* p.S1029C). This patient presented to psychiatric services aged 73 with prominent visual hallucinations which had become distressing, they had begun approximately 1 year earlier. She was also noted to be quite disinhibited and aggressive. A mental test score was 25/37 on presentation aged 73. Cognitive symptoms began to fluctuate markedly over the next few years, and 4 years later she developed moderate parkinsonism. The patient died 5 years after onset aged 78.

Neuropathological examination, as outlined in Table 2 (main text) showed an NFT Braak stage of 5, Aβ Phase of 4, neocortical Lewy body disease/LB Braak stage of 6, CAA stage of 2 and no TDP-43 positive intracytoplasmic inclusions.

Patient H (*GIGYF2* p.S66T). This patient was referred aged 88 with a 3 month history of poor short term memory, worsening self care and frontal behaviour. Her Mini Mental State Examination (MMSE) was 21/30 at this point. She deteriorated rapidly over the next 9 months and died aged 89 one year after symptom onset. No motor features developed at any point.

Neuropathological examination, as outlined in Table 2 (main text) showed an NFT Braak stage of 4, Aβ Phase of 3, neocortical Lewy body disease/LB Braak stage of 5, CAA stage of 2 and no TDP-43 positive intracytoplasmic inclusions.

**Discussion of variants unlikely to be pathogenic**

The p.R71W mutation in the *PSEN-2* gene was present in one patient, and whilst identified in at least 3 previous families with AD has not segregated with disease in all cases [^45-47^](#_ENREF_45). Three patients also carried rare mutations in *GRN* (p.A324T, and p.R433W). The p.A324T and p.433W mutations do not affect cerebral granulin expression [^48^](#_ENREF_48), and the p.A324T mutation has previously been seen in a similar frequency in patients and controls [^49^](#_ENREF_49).

In the *EIF4G1* gene, the p.L1038P variant has a MAF of 0.03 or greater in both 1000G and ESP6400, and therefore a relatively common polymorphism. The p.T634S mutation was seen in one patient and no controls, and has a MAF of 0.000154 in 1000G. This mutation to our knowledge has not been described in any neurodegenerative disease before. However, this mutation sits between the EIF4E and EIF4A domains, and previous mutations in this region have been non-pathogenic [^50^](#_ENREF_50). The mutation is also predicted to be benign and tolerated by PolyPhen2 and LIB Sift respectively (Table S3). A single patient had the p.H1165R in the *GIGY2F* gene. This variant is unlikely to be pathogenic having only previously been reported in control patients as the p.H1171R variant [^51^](#_ENREF_51) and p.H1192R variant [^52^](#_ENREF_52). In the *HTRA2* gene, the p.G399S mutation was seen in a single patient and a single control. This mutation was originally suggested as a highly penetrant allele [^26^](#_ENREF_26), however follow up studies have failed to replicate this association, showing equal prevalence in patients and controls [^53^](#_ENREF_53)^,^ [^54^](#_ENREF_54). Similarly, an equal prevalence of the *HTRA2* p.A141S mutation also reported between cases and controls in previous candidate gene studies [^53^](#_ENREF_53)^,^ [^54^](#_ENREF_54). The p.L72P *HTRA2* mutation has not previously been described in candidate gene studies, and whilst predicted by 2 of the 3 prediction software programmes to be damaging, it was also seen in a single control, suggesting it is unlikely to be pathogenic.

A p.K154E mutation in *SQSTM1* was identified in one patient. This mutation is within the ZZ-domain of the protein, a region in which pathogenic mutations have not previously been described. In addition, it was only predicted to be deleterious by one of three software prediction programmes.

The single homozygous mutation seen in a patient was the p.Q115L mutation in *PINK1*. This mutation was previously described in a neurologically normal control patient has not been seen in patients and is therefore unlikely to be pathogenic [^55^](#_ENREF_55).

| **Gene** | **Ch** | **Position** | **R/V** | **dbSNP 135** | **Protein change** | **Phenotype** | **SIFT** | **PolyPhen2** | **Mutation-taster** | **CADD score** | **ESP 6500** | **1000G** | **ExAC** | **Number of patients covered** | **Cases** | **Number of controls covered** | **Control patients** |
| --- | --- | --- | --- | --- | --- | --- | --- | --- | --- | --- | --- | --- | --- | --- | --- | --- | --- |
| ***GIGYF2*** | 2 | 233712109 | A/G | rs72554081 | p.H1165R | U | NA | NA | D | 23.4 | 0.00050 | 0.00146 | 0.00157 | 87 | 2 | 93 | 0 |
| ***GIGYF2*** | 2 | 233655834 | T/A | rs148277228 | p.D131E | U | T | B | N | 10.9 | 0.00092 |  | 0.000454 | 86 | 1 | 92 | 0 |
| ***PSEN-2*** | 1 | 227071475 | C/T | rs140501902 | p.R71W | U | D | P | N | 26.2 | 0.00298 | 0.00230 | 0.00335 | 86 | 1 | 90 | 0 |
| ***GRN*** | 17 | 42428954 | G/A | rs63750541 | p.A324T | U | T | B | D | 0.180 | 0.00138 |  | 0.000850 | 83 | 2 | 90 | 1 |
| ***GRN*** | 17 | 42429500 | C/T | rs63750412 | p.R433W | U | D | P | N | 25.2 | 0.00223 | 0.00270 | 0.00433 | 85 | 1 | 93 | 0 |
| ***EIF4G1*** | 3 | 184042001 | A/T | rs111500185 | p.T634S | U | T | B | D | 15.2 |  | 0.00015 | 0.0000330 | 87 | 1 | 93 | 0 |
| ***EIF4G1*** | 3 | 184045410 | T/C | rs2230570 | p.L1038P | U | T | B | P | 0.654 | 0.03000 | 0.04190 | 0.0226 | 82 | 1 | 92 | 5 |
| ***HTRA2*** | 2 | 74757554 | G/T | rs72470544 | p.A141S | U | T | B | N | 0.008 | 0.01681 | 0.02000 | 0.0266 | 83 | 5 | 91 | 4 |
| ***HTRA2*** | 2 | 74757348 | T/C | rs150047108 | p.L72P | U | D | D | N | 10.7 | 0.00140 | 0.00216 | 0.00270 | 86 | 1 | 90 | 1 |
| ***HTRA2*** | **2** | 74759825 | G/A | rs72470545 | p.G399S | PD | D | P | D | 24.1 | 0.00363 | 0.00230 | 0.00438 | 87 | 1 | 93 | 1 |
| ***SQSTM1*** | 5 | 179252184 | A/G | rs11548633 | p.K154E | U | T | D | N | 24.4 | 0.00261 | 0.00320 | 0.00242 | 87 | 2 | 92 | 0 |
| **Homozygous variants** | | | | |  |  |  |  |  |  |  |  |  |  |  |  |  |
|  |  |  |  |  |  |  |  |  |  |  |  |  |  |  |  |  |  |
| ***PINK1*** | 1 | 20960385 | A/T* | rs148871409 | p.Q115L | NRF |  |  | D | 17.0 | 0.020 | 0.033 | 0.0643 | 86 | 1 | 91 | 0 |

**Supplementary tables**

**Supplementary Table 1**. Rare protein altering heterozygous and homozygous non-synonymous variants in genes known to cause monogenic forms of neurodegenerative disease (PD, AD, FTD-ALS) which are unlikely to be pathogenic in the 91 DLB cases studied here. The number of patients covered at 30 fold sequence depth, and the number of case and control patients carrying each mutation is described. Key: Ch; Chromosome, Phenotype; U – Unknown or not described. NRF – Non-risk factor. ESP6500 [^56^](#_ENREF_56) , 1000G [^57^](#_ENREF_57) and ExAC [^58^](#_ENREF_58) – allele frequencies in the respective databases.

| *Gene* | Ch | Position | R/V | dbSNP135 | Protein change | Phenotype | SIFT | PolyPhen2 | Mutation-Taster | CADD score | ESP6500 | 1000G | ExAC | Number cases covered | Cases | Number controls covered | Control patients | P-Value |
| --- | --- | --- | --- | --- | --- | --- | --- | --- | --- | --- | --- | --- | --- | --- | --- | --- | --- | --- |
| ***LRRK2*** | 12 | 40707861 | C/T | rs33958906 | p.P1542S | U | D | D | D | 28.7 | 0.02343 | 0.02000 | 0.0301 | 85 | 9 | 93 | 5 | 0.16 |
|  |  | 40713899 | T/C | rs35303786 | p.M1646T | U | T | D | D | 17.9 | 0.01136 | 0.01000 | 0.00916 | 87 | 4 | 92 | 3 | 0.47 |
|  |  | 40740686 | A/G | rs33995883 | p.N2081D | U | T | P | D | 24.7 | 0.01000 | 0.01353 | 0.0176 | 87 | 1 | 92 | 4 | 0.20 |
|  |  | 40645148 | C/T | rs141262110 | p.T358M | U | D | B | N | 14.8 |  | 0.00015 | 0.0000748 | 87 | 1 | 91 | 0 | 0.49 |
|  |  | 40653336 | G/T | NA | p.M239I | U | T | P | N | 23.5 | 0.00009 |  | 0.0000165 | 86 | 1 | 93 | 0 | 0.48 |
| ***SCARB2*** | 4 | 77089557 | C/T | rs77814624 | p.V253I | U | T | B | N | 0.397 | 0.02553 | 0.02000 | 0.00754 | 87 | 0 | 93 | 1 | 1.00 |
|  |  | 77100807 | T/C | rs143655258 | p.M159V | U | T | B | N | 0.078 | 0.00861 | 0.01000 | 0.00896 | 87 | 1 | 93 | 2 | 1.00 |
|  |  | 77100837 | C/T | rs147159813 | p.V149M | U | T | B | N | 5.67 | 0.00246 | 0.00180 | 0.00205 | 87 | 1 | 93 | 2 | 1.00 |
|  |  | 77116941 | T/C | rs138955932 | p.Y65C | U | D | D | D | 25.3 | 0.00008 |  | 0.000330 | 87 | 0 | 93 | 1 | 1.00 |
| ***GBA*** | 1 | 155206037 | G/A | rs75548401 | p.T295M | U | T | B | D | 22.2 | 0.00502 | 0.00320 | 0.00657 | 83 | 2 | 92 | 0 | 0.23 |
|  |  | 155206167 | C/T | rs2230288 | p.E252K | PD | T | B | D | 17.3 | 0.00883 | 0.01000 | 0.00979 | 86 | 3 | 93 | 3 | 0.62 |
|  |  | 155205043 | A/G | rs421016 | p.L370P | PD | D | P | D | 24.8 | 0.00037 |  | 0.00310 | 85 | 2 | 93 | 0 | 0.23 |
|  |  | 155205634 | T/C | rs76763715 | p.N296S | PD | T | D | D | 22.7 | 0.00205 | 0.00140 | 0.00221 | 87 | 2 | 93 | 0 | 0.23 |
|  |  | 155210420 | C/T | Splice site changes | | U |  | | | 25.0 | 0.00000 | 0.00000 | 0.00000 | 87 | 1 | 93 | 0 | 0.48 |

**Supplementary Table 2.** Rare exonic non-synonymous variants in *GBA* and *LRRK2* detected in the 91 DLB cases. All variants were covered at 30-fold depth in at least 80% of cases and controls. The number of patients covered at 30-fold sequence depth, and the number of case and control patients carrying each mutation is described. Key: Ch; Chromosome, Phenotype; AD – Alzheimer’s disease, FTD – Frontotemporal dementia – Amyotrophic lateral sclerosis, PD – Parkinson’s disease, U – Unknown or not described. ESP6500 [^56^](#_ENREF_56) ,1000G [^57^](#_ENREF_57) and ExAC [^58^](#_ENREF_58) – allele frequency in the respective databases. P-value reflects the difference for each allele frequency between the patient and control group as performed by Fisher’s exact test.

| ***Gene*** | **ch** | **Position** | **R/V** | **dbSNP135** | **Protein change** | **Pheno-type** | **SIFT** | **Poly-phen2** | **Mutation taster** | **CADD score** | **ESP6500** | **1000G** | **ExAC** | **Number of patients covered** | **Number of cases** | **Number of controls covered** | **Control patients** | **Fisher's p value (2-tail)** |
| --- | --- | --- | --- | --- | --- | --- | --- | --- | --- | --- | --- | --- | --- | --- | --- | --- | --- | --- |
| ***PARK2*** | 6 | 161781225 | C/T | rs1801334 | p.D394N | RF | T | B | D | 23.0 | 0.03188 | 0.02 | 0.0256 | 87 | 9 | 91 | 9 | 0.56 |
|  |  | 162864410 | delCT |  | Stop | U | D | D | D | NA | 0 | 0 | 0 | 86 | 1 | 90 | 0 | 0.47 |
| ***PARK7*** | 1 | 8030994 | G/A | rs71653619 | p.R98Q | U | T | B | D | 13.6 | 0.00818 | 0.01 | 0.00815 | 85 | 3 | 93 | 3 | 0.61 |
| ***PLA2G6*** | 22 | 38528944 | G/A |  | p.A51V | U | T | D | D | 23.8 |  |  | 0.00004513 | 83 | 1 | 92 | 0 | 0.47 |
| ***ATP13A2*** | 1 | 17312586 | G/A | rs189334432 | p.P364L | U | D | D | N | 12.7 |  | 0.0023 | 0.00521 | 87 | 1 | 93 | 1 | 0.73 |
|  |  | 17312592 | G/A | rs15786 | p.P362L | RF | D | B | N | 15.4 | 0.04315 | 0.02 | 0.0794 | 86 | 13 | 93 | 12 | 0.42 |
|  |  | 17312596 | T/A | rs41273151 | p.T361S | U | D | B | N | 17.4 | 0.00329 | 0.0037 | 0.00649 | 86 | 1 | 93 | 2 | 0.53 |
|  |  | 17322793 | G/C | rs149372969 | p.L151V | U | T | B | N | 5.48 | 0.00131 | 0.0009 | 0.00117 | 86 | 2 | 92 | 1 | 0.48 |

**Supplementary Table 3.** All heterozygous variants in genes known to cause monogenic forms of disease in the homozygous state. Non-synonymous variants, with a mean allele frequency of < 0.05 in the 1000G, ESP6500 and/or in-house local exome database covered at 30-fold depth in at least 80% of cases and controls were selected. Heterozygous variants in genes known to cause autosomal recessive forms of AD, PD or FTD (see table 1 – main text) were selected. The number of patients covered at 30-fold sequence depth, and the number of case and control patients carrying each mutation is described. Key: Ch; Chromosome, Phenotype; AD – Alzheimer’s disease, FTD – Frontotemporal dementia – Amyotrophic lateral sclerosis, PD – Parkinson’s disease, RF – Suggested risk factor for PD, U – Unknown or not described. ESP6500 [^56^](#_ENREF_56) , 1000G [^57^](#_ENREF_57) and ExAC [^58^](#_ENREF_58) – allele frequency in the respective databases. P-value reflects the difference for each allele frequency between the patient and control group as performed by Fisher’s exact test.

**Supplementary References**

1. Langmead B, Trapnell C, Pop M, Salzberg SL. Ultrafast and memory-efficient alignment of short DNA sequences to the human genome. Genome biology. 2009;10(3):R25. PubMed PMID: 19261174. Pubmed Central PMCID: 2690996. Epub 2009/03/06. eng.

2. Li H, Handsaker B, Wysoker A, Fennell T, Ruan J, Homer N, et al. The Sequence Alignment/Map format and SAMtools. Bioinformatics. 2009 Aug 15;25(16):2078-9. PubMed PMID: 19505943. Pubmed Central PMCID: 2723002.

3. Koboldt DC, Chen K, Wylie T, Larson DE, McLellan MD, Mardis ER, et al. VarScan: variant detection in massively parallel sequencing of individual and pooled samples. Bioinformatics. 2009 Sep 1;25(17):2283-5. PubMed PMID: 19542151. Pubmed Central PMCID: 2734323. Epub 2009/06/23. eng.

4. Albers CA, Lunter G, MacArthur DG, McVean G, Ouwehand WH, Durbin R. Dindel: accurate indel calls from short-read data. Genome Res. 2011 Jun;21(6):961-73. PubMed PMID: 20980555. Pubmed Central PMCID: 3106329. Epub 2010/10/29. eng.

5. Schwarz JM, Rodelsperger C, Schuelke M, Seelow D. MutationTaster evaluates disease-causing potential of sequence alterations. Nat Methods. 2010 Aug;7(8):575-6. PubMed PMID: 20676075. Epub 2010/08/03. eng.

6. Kumar P, Henikoff S, Ng PC. Predicting the effects of coding non-synonymous variants on protein function using the SIFT algorithm. Nat Protoc. 2009;4(7):1073-81. PubMed PMID: 19561590.

7. Adzhubei IA, Schmidt S, Peshkin L, Ramensky VE, Gerasimova A, Bork P, et al. A method and server for predicting damaging missense mutations. Nat Methods. 2010 Apr;7(4):248-9. PubMed PMID: 20354512. Pubmed Central PMCID: 2855889.

8. Kircher M, Witten DM, Jain P, O'Roak BJ, Cooper GM, Shendure J. A general framework for estimating the relative pathogenicity of human genetic variants. Nature genetics. 2014 Mar;46(3):310-5. PubMed PMID: 24487276. Pubmed Central PMCID: 3992975.

9. Polymeropoulos MH, Higgins JJ, Golbe LI, Johnson WG, Ide SE, Di Iorio G, et al. Mapping of a gene for Parkinson's disease to chromosome 4q21-q23. Science. 1996 Nov 15;274(5290):1197-9. PubMed PMID: 8895469.

10. Polymeropoulos MH, Lavedan C, Leroy E, Ide SE, Dehejia A, Dutra A, et al. Mutation in the alpha-synuclein gene identified in families with Parkinson's disease. Science. 1997 Jun 27;276(5321):2045-7. PubMed PMID: 9197268. Epub 1997/06/27. eng.

11. Funayama M, Hasegawa K, Kowa H, Saito M, Tsuji S, Obata F. A new locus for Parkinson's disease (PARK8) maps to chromosome 12p11.2-q13.1. Ann Neurol. 2002 Mar;51(3):296-301. PubMed PMID: 11891824.

12. Renton AE, Majounie E, Waite A, Simon-Sanchez J, Rollinson S, Gibbs JR, et al. A hexanucleotide repeat expansion in C9ORF72 is the cause of chromosome 9p21-linked ALS-FTD. Neuron. 2011 Oct 20;72(2):257-68. PubMed PMID: 21944779. Pubmed Central PMCID: 3200438.

13. Rosen DR, Siddique T, Patterson D, Figlewicz DA, Sapp P, Hentati A, et al. Mutations in Cu/Zn superoxide dismutase gene are associated with familial amyotrophic lateral sclerosis. Nature. 1993 Mar 4;362(6415):59-62. PubMed PMID: 8446170.

14. Rogaev EI, Sherrington R, Rogaeva EA, Levesque G, Ikeda M, Liang Y, et al. Familial Alzheimer's disease in kindreds with missense mutations in a gene on chromosome 1 related to the Alzheimer's disease type 3 gene. Nature. 1995 Aug 31;376(6543):775-8. PubMed PMID: 7651536. Epub 1995/08/31. eng.

15. Levy-Lahad E, Wasco W, Poorkaj P, Romano DM, Oshima J, Pettingell WH, et al. Candidate gene for the chromosome 1 familial Alzheimer's disease locus. Science. 1995 Aug 18;269(5226):973-7. PubMed PMID: 7638622. Epub 1995/08/18. eng.

16. Goate A, Chartier-Harlin MC, Mullan M, Brown J, Crawford F, Fidani L, et al. Segregation of a missense mutation in the amyloid precursor protein gene with familial Alzheimer's disease. Nature. 1991 Feb 21;349(6311):704-6. PubMed PMID: 1671712. Epub 1991/02/21. eng.

17. Cruts M, Gijselinck I, van der Zee J, Engelborghs S, Wils H, Pirici D, et al. Null mutations in progranulin cause ubiquitin-positive frontotemporal dementia linked to chromosome 17q21. Nature. 2006 Aug 24;442(7105):920-4. PubMed PMID: 16862115.

18. Dumanchin C, Camuzat A, Campion D, Verpillat P, Hannequin D, Dubois B, et al. Segregation of a missense mutation in the microtubule-associated protein tau gene with familial frontotemporal dementia and parkinsonism. Human molecular genetics. 1998 Oct;7(11):1825-9. PubMed PMID: 9736786.

19. Skibinski G, Parkinson NJ, Brown JM, Chakrabarti L, Lloyd SL, Hummerich H, et al. Mutations in the endosomal ESCRTIII-complex subunit CHMP2B in frontotemporal dementia. Nat Genet. 2005 Aug;37(8):806-8. PubMed PMID: 16041373.

20. Kwiatkowski TJ, Jr., Bosco DA, Leclerc AL, Tamrazian E, Vanderburg CR, Russ C, et al. Mutations in the FUS/TLS gene on chromosome 16 cause familial amyotrophic lateral sclerosis. Science. 2009 Feb 27;323(5918):1205-8. PubMed PMID: 19251627.

21. Greenway MJ, Andersen PM, Russ C, Ennis S, Cashman S, Donaghy C, et al. ANG mutations segregate with familial and 'sporadic' amyotrophic lateral sclerosis. Nat Genet. 2006 Apr;38(4):411-3. PubMed PMID: 16501576.

22. Johnson JO, Mandrioli J, Benatar M, Abramzon Y, Van Deerlin VM, Trojanowski JQ, et al. Exome sequencing reveals VCP mutations as a cause of familial ALS. Neuron. 2010 Dec 9;68(5):857-64. PubMed PMID: 21145000. Pubmed Central PMCID: 3032425.

23. Kwok CT, Morris A, de Belleroche JS. Sequestosome-1 (SQSTM1) sequence variants in ALS cases in the UK: prevalence and coexistence of SQSTM1 mutations in ALS kindred with PDB. Eur J Hum Genet. 2013 Aug 14. PubMed PMID: 23942205.

24. Leroy E, Boyer R, Auburger G, Leube B, Ulm G, Mezey E, et al. The ubiquitin pathway in Parkinson's disease. Nature. 1998 Oct 1;395(6701):451-2. PubMed PMID: 9774100.

25. Pankratz N, Nichols WC, Uniacke SK, Halter C, Rudolph A, Shults C, et al. Significant linkage of Parkinson disease to chromosome 2q36-37. Am J Hum Genet. 2003 Apr;72(4):1053-7. PubMed PMID: 12638082. Pubmed Central PMCID: 1180337.

26. Strauss KM, Martins LM, Plun-Favreau H, Marx FP, Kautzmann S, Berg D, et al. Loss of function mutations in the gene encoding Omi/HtrA2 in Parkinson's disease. Human molecular genetics. 2005 Aug 1;14(15):2099-111. PubMed PMID: 15961413.

27. Lucking CB, Durr A, Bonifati V, Vaughan J, De Michele G, Gasser T, et al. Association between early-onset Parkinson's disease and mutations in the parkin gene. N Engl J Med. 2000 May 25;342(21):1560-7. PubMed PMID: 10824074.

28. Valente EM, Abou-Sleiman PM, Caputo V, Muqit MM, Harvey K, Gispert S, et al. Hereditary early-onset Parkinson's disease caused by mutations in PINK1. Science. 2004 May 21;304(5674):1158-60. PubMed PMID: 15087508.

29. Bonifati V, Rizzu P, van Baren MJ, Schaap O, Breedveld GJ, Krieger E, et al. Mutations in the DJ-1 gene associated with autosomal recessive early-onset parkinsonism. Science. 2003 Jan 10;299(5604):256-9. PubMed PMID: 12446870.

30. Ramirez A, Heimbach A, Grundemann J, Stiller B, Hampshire D, Cid LP, et al. Hereditary parkinsonism with dementia is caused by mutations in ATP13A2, encoding a lysosomal type 5 P-type ATPase. Nat Genet. 2006 Oct;38(10):1184-91. PubMed PMID: 16964263.

31. Paisan-Ruiz C, Bhatia KP, Li A, Hernandez D, Davis M, Wood NW, et al. Characterization of PLA2G6 as a locus for dystonia-parkinsonism. Ann Neurol. 2009 Jan;65(1):19-23. PubMed PMID: 18570303.

32. Shojaee S, Sina F, Banihosseini SS, Kazemi MH, Kalhor R, Shahidi GA, et al. Genome-wide linkage analysis of a Parkinsonian-pyramidal syndrome pedigree by 500 K SNP arrays. Am J Hum Genet. 2008 Jun;82(6):1375-84. PubMed PMID: 18513678. Pubmed Central PMCID: 2427312.

33. Chapman J, Estupinan J, Asherov A, Goldfarb LG. A simple and efficient method for apolipoprotein E genotype determination. Neurology. 1996 May;46(5):1484-5. PubMed PMID: 8628509.

34. Braak H, Alafuzoff I, Arzberger T, Kretzschmar H, Del Tredici K. Staging of Alzheimer disease-associated neurofibrillary pathology using paraffin sections and immunocytochemistry. Acta neuropathologica. 2006 Oct;112(4):389-404. PubMed PMID: 16906426. Pubmed Central PMCID: 3906709.

35. Alafuzoff I, Ince PG, Arzberger T, Al-Sarraj S, Bell J, Bodi I, et al. Staging/typing of Lewy body related alpha-synuclein pathology: a study of the BrainNet Europe Consortium. Acta Neuropathol. 2009 Jun;117(6):635-52. PubMed PMID: 19330340.

36. Thal DR, Rub U, Orantes M, Braak H. Phases of A beta-deposition in the human brain and its relevance for the development of AD. Neurology. 2002 Jun 25;58(12):1791-800. PubMed PMID: 12084879.

37. Thal DR, Ghebremedhin E, Orantes M, Wiestler OD. Vascular pathology in Alzheimer disease: correlation of cerebral amyloid angiopathy and arteriosclerosis/lipohyalinosis with cognitive decline. J Neuropathol Exp Neurol. 2003 Dec;62(12):1287-301. PubMed PMID: 14692704.

38. Lleo A, Blesa R, Gendre J, Castellvi M, Pastor P, Queralt R, et al. A novel presenilin 2 gene mutation (D439A) in a patient with early-onset Alzheimer's disease. Neurology. 2001 Nov 27;57(10):1926-8. PubMed PMID: 11723295.

39. Marcon G, Giaccone G, Cupidi C, Balestrieri M, Beltrami CA, Finato N, et al. Neuropathological and clinical phenotype of an Italian Alzheimer family with M239V mutation of presenilin 2 gene. J Neuropathol Exp Neurol. 2004 Mar;63(3):199-209. PubMed PMID: 15055444.

40. Parkinson N, Ince PG, Smith MO, Highley R, Skibinski G, Andersen PM, et al. ALS phenotypes with mutations in CHMP2B (charged multivesicular body protein 2B). Neurology. 2006 Sep 26;67(6):1074-7. PubMed PMID: 16807408.

41. Fecto F, Yan J, Vemula SP, Liu E, Yang Y, Chen W, et al. SQSTM1 mutations in familial and sporadic amyotrophic lateral sclerosis. Arch Neurol. 2011 Nov;68(11):1440-6. PubMed PMID: 22084127.

42. Le Ber I, Camuzat A, Guerreiro R, Bouya-Ahmed K, Bras J, Nicolas G, et al. SQSTM1 Mutations in French Patients With Frontotemporal Dementia or Frontotemporal Dementia With Amyotrophic Lateral Sclerosis. JAMA Neurol. 2013 Nov 1;70(11):1403-10. PubMed PMID: 24042580.

43. Klein C, Djarmati A, Hedrich K, Schafer N, Scaglione C, Marchese R, et al. PINK1, Parkin, and DJ-1 mutations in Italian patients with early-onset parkinsonism. Eur J Hum Genet. 2005 Sep;13(9):1086-93. PubMed PMID: 15970950.

44. Sun M, Latourelle JC, Wooten GF, Lew MF, Klein C, Shill HA, et al. Influence of heterozygosity for parkin mutation on onset age in familial Parkinson disease: the GenePD study. Arch Neurol. 2006 Jun;63(6):826-32. PubMed PMID: 16769863.

45. Brouwers N, Sleegers K, Van Broeckhoven C. Molecular genetics of Alzheimer's disease: an update. Ann Med. 2008;40(8):562-83. PubMed PMID: 18608129.

46. Guerreiro RJ, Baquero M, Blesa R, Boada M, Bras JM, Bullido MJ, et al. Genetic screening of Alzheimer's disease genes in Iberian and African samples yields novel mutations in presenilins and APP. Neurobiology of aging. 2010 May;31(5):725-31. PubMed PMID: 18667258. Pubmed Central PMCID: 2850052.

47. Sleegers K, Roks G, Theuns J, Aulchenko YS, Rademakers R, Cruts M, et al. Familial clustering and genetic risk for dementia in a genetically isolated Dutch population. Brain. 2004 Jul;127(Pt 7):1641-9. PubMed PMID: 15130954.

48. Finch N, Baker M, Crook R, Swanson K, Kuntz K, Surtees R, et al. Plasma progranulin levels predict progranulin mutation status in frontotemporal dementia patients and asymptomatic family members. Brain. 2009 Mar;132(Pt 3):583-91. PubMed PMID: 19158106. Pubmed Central PMCID: 2664450.

49. Yu CE, Bird TD, Bekris LM, Montine TJ, Leverenz JB, Steinbart E, et al. The spectrum of mutations in progranulin: a collaborative study screening 545 cases of neurodegeneration. Arch Neurol. 2010 Feb;67(2):161-70. PubMed PMID: 20142524. Pubmed Central PMCID: 2901991.

50. Nuytemans K, Bademci G, Inchausti V, Dressen A, Kinnamon DD, Mehta A, et al. Whole exome sequencing of rare variants in EIF4G1 and VPS35 in Parkinson disease. Neurology. 2013 Mar 12;80(11):982-9. PubMed PMID: 23408866. Pubmed Central PMCID: 3653206.

51. Lautier C, Goldwurm S, Durr A, Giovannone B, Tsiaras WG, Pezzoli G, et al. Mutations in the GIGYF2 (TNRC15) gene at the PARK11 locus in familial Parkinson disease. Am J Hum Genet. 2008 Apr;82(4):822-33. PubMed PMID: 18358451. Pubmed Central PMCID: 2427211.

52. Bras J, Simon-Sanchez J, Federoff M, Morgadinho A, Januario C, Ribeiro M, et al. Lack of replication of association between GIGYF2 variants and Parkinson disease. Human molecular genetics. 2009 Jan 15;18(2):341-6. PubMed PMID: 18923002. Pubmed Central PMCID: 2638775.

53. Simon-Sanchez J, Singleton AB. Sequencing analysis of OMI/HTRA2 shows previously reported pathogenic mutations in neurologically normal controls. Human molecular genetics. 2008 Jul 1;17(13):1988-93. PubMed PMID: 18364387. Pubmed Central PMCID: 2574854.

54. Ross OA, Soto AI, Vilarino-Guell C, Heckman MG, Diehl NN, Hulihan MM, et al. Genetic variation of Omi/HtrA2 and Parkinson's disease. Parkinsonism Relat Disord. 2008 Nov;14(7):539-43. PubMed PMID: 18790661. Pubmed Central PMCID: 2614082.

55. Ishihara-Paul L, Hulihan MM, Kachergus J, Upmanyu R, Warren L, Amouri R, et al. PINK1 mutations and parkinsonism. Neurology. 2008 Sep 16;71(12):896-902. PubMed PMID: 18685134. Pubmed Central PMCID: 2676945.

56. Hao H, Moraes CT. Functional and molecular mitochondrial abnormalities associated with a C --> T transition at position 3256 of the human mitochondrial genome. The effects of a pathogenic mitochondrial tRNA point mutation in organelle translation and RNA processing. J Biol Chem. 1996;271(4):2347-52.

57. Genomes Project C, Abecasis GR, Auton A, Brooks LD, DePristo MA, Durbin RM, et al. An integrated map of genetic variation from 1,092 human genomes. Nature. 2012 Nov 1;491(7422):56-65. PubMed PMID: 23128226. Pubmed Central PMCID: 3498066.

58. Exome Aggregation Consortium (ExAC) Cambridge, MA, USA [cited 2015 September]. Available from: <http://exac.broadinstitute.org>.
